# Manipulation of the microbiome in critical illness—probiotics as a preventive measure against ventilator-associated pneumonia

**DOI:** 10.1186/s40635-019-0238-1

**Published:** 2019-07-25

**Authors:** Marel C. E. van Ruissen, Lieuwe D. Bos, Robert P. Dickson, Arjen M. Dondorp, Constance Schultsz, Marcus J. Schultz

**Affiliations:** 1 Amsterdam Institute for Global Health and Development (AIGHD), Academic Medical Center, Amsterdam, The Netherlands; 2 Department of Pulmonology, Academic Medical Center, Amsterdam, The Netherlands; 3 Department of Intensive Care, Academic Medical Center, C3-425, Meibergdreef 9, 1105 AZ Amsterdam, The Netherlands; 4 Department of Internal Medicine, Division of Pulmonary and Critical Care Medicine, University of Michigan Medical School, Ann Arbor, MI USA; 5 Mahidol Oxford Tropical Medicine Research Unit (MORU), Mahidol University, Bangkok, Thailand; 6 Laboratory of Experimental Intensive Care and Anesthesiology (L·E·I·C·A), Academic Medical Center, Amsterdam, The Netherlands

**Keywords:** VAP, Microbiome, Probiotics, Prevention

## Abstract

**Objective:**

To describe the possible modes of action of probiotics and provide a systematic review of the current evidence on the efficacy of probiotics to prevent ventilator-associated pneumonia (VAP) in critically ill patients.

**Methods:**

We conducted an unrestricted search of the English language medical literature. For each individual study, the relative risk of VAP was calculated using the reported primary outcome data.

**Results:**

The search identified a total of 72 articles. Eight articles enrolling a total of 1229 patients fulfilled the inclusion and exclusion criteria. In four trials, the investigators were blinded for the intervention, and two trials used an intention-to-treat analysis. Loss to follow-up with regard to the primary endpoint ranged from 0 to 14% in the intervention groups and from 0 to 16% in the control groups. The incidence of VAP expressed as the percentage of studied patients was reported in seven trials. The incidence of VAP ranged from 4 to 36% in the intervention groups and from 13 to 50% in the control groups. The relative risk for VAP ranged between 0.30 and 1.41. Three trials showed a significant difference in favor of probiotic therapy between the intervention and the control groups.

**Conclusions:**

The incidence of VAP tended to be lower in patients treated with probiotics in most trials identified by the systematic search. Due to the heterogeneity of the studies and the low quality of evidence, it remains difficult to draw firm conclusions. The efficacy of preventive probiotics should be studied in more detail in future trials. Application of probiotics for the prevention of VAP seems to be safe with only few side effects reported in the selected trials.

**Electronic supplementary material:**

The online version of this article (10.1186/s40635-019-0238-1) contains supplementary material, which is available to authorized users.

## Background

Critically ill patients receiving mechanical ventilation are at risk of developing ventilator-associated pneumonia (VAP). VAP accounts for around a third of all hospital-acquired infections in the intensive care unit (ICU), which translates into an estimated 29% VAP attributable ICU mortality [1]. Although reliable data are scarce, the incidence of VAP is likely to be higher in low- and middle-income countries (LMICs). A study from India reported a VAP incidence of 38% (40 episodes per 1000 ventilation days) [2]. Prevention of VAP thus has great potential to reduce ICU mortality, but also to reduce antibiotic use, which is an important driver of antimicrobial drug resistance.

Stringent implementation of “infection prevention and control” practices is essential for reducing VAP incidences, including hand hygiene measures, appropriate use of gloves, use of clean tubing and suction sets, comprehensive oral care, and limiting the exchange of equipment and staff between individual patients. In addition, elevation of the head of the bed, avoiding gastric overdistention, sufficient endotracheal cuff pressure, and frequent assessment of readiness to wean are important measures. Implementation requires human and non-human resources, training and surveillance, and is often incomplete, in particular in a resource-limited ICU setting [3].

Micro-aspiration and colonization with more pathogenic bacterial flora are important pathogenic mechanisms in VAP. Oral and enteral application of antibiotics throughout admission in the ICU, deployed as either selective digestive tract decontamination (SDD) or selective oral decontamination (SOD), have shown to reduce VAP incidence and reduce ICU mortality [4–6]. The presumed mode of action is through the promotion of “colonization resistance” against opportunistic healthcare-associated microorganisms [7]. An alternative approach promoting colonization resistance is manipulation of the microbiome through the use of probiotics. Compared to SDD and SOD, this approach avoids additional antimicrobial drug pressure and is independent of prevailing antimicrobial drug resistance. Problems with antimicrobial drug resistance are increasing, in particular in resource-limited settings, which include antimicrobials used in SDD. Probiotics should also be differentiated from prebiotics; these are metabolic compounds administrated to an individual to change the growth of already microbes that are already present in the body.

The current study discusses the possible modes of action of probiotics and provides a systematic review of the current evidence on the efficacy of probiotics to prevent VAP in adult ICU patients.

### An ecologic rationale for probiotics

#### The microbiome

Until recently, the notion of “lung ecology” was a contradiction in terms. For more than a century following the dawn of germ theory, textbooks taught that “the normal lung is free from bacteria.” Thus, pneumonia represented the invasion of a sterile space by a microbial inoculum large enough to overwhelm host defenses. This conceptual model of pneumonia pathogenesis had no need for the concepts of microbial ecology; the lungs no more represented an ecosystem than does a sterilized flask of culture media. Yet in the past decade, we have learned that the lungs, even in health, contain a dynamic ecosystem of diverse bacterial communities. The use of culture-independent techniques of microbial identification, most often amplicon sequencing of the bacterial 16S rRNA gene, has taught us that the lungs harbor their own “microbiome,” detectable in health, altered in disease, and predictive of clinical outcomes, even in non-infectious lung disease [8].

#### Ecology

The discovery of the lung microbiome has prompted reconsideration of our conceptual models of the pathogenesis of VAP [9]. Whereas for decades, investigators have studied the *microbiologic* and *immunologic* causes of VAP; we must now consider pneumonia as an *ecologic* phenomenon. Ecologically, pneumonia represents an abrupt drop in respiratory microbial diversity with a similarly abrupt increase in microbial burden. To an ecologist, pneumonia more closely resembles a freshwater algal bloom than it does the inoculation of a culture flask: a sudden, cataclysmic drop in community diversity, dominated by an emergent pathogen. Thus, to understand the rationale for probiotics in VAP, we need to consider the ecologic forces that determine the composition of lung microbiota. Like any community, the lung microbiome is determined by a balance of three ecologic forces: immigration, elimination, and the relative reproduction rates of community members. If the population of lung bacteria is altered in a disease state, it must be attributable to some combination of altered immigration, e.g., via oropharyngeal aspiration, altered elimination, e.g., via impaired mucociliary clearance, or environmental pressure favoring the relative growth of select bacteria, e.g., via altered abundance of nutritional substrate. By the same argument, if probiotic therapy is *protective* against VAP, it must work via one of these three ecologic mechanisms.

#### Immigration of the upper gastrointestinal tract

The primary source community of bacteria to the lung microbiome, both in health and disease, is the oropharynx. Microaspiration of oropharyngeal secretions, ubiquitous but subclinical in health, is dramatically accelerated by sedation and endotracheal intubation [10, 11]. Thus, the microbial composition of the upper gastrointestinal tract is a key determinant of the lung microbiome, especially in critical illness. In critically ill patients, “normal” pharyngeal bacteria are displaced within days by VAP-associated bacteria with pathogenic potential [12, 13]. Thus, one potential ecologic mechanism of benefit for enteric probiotics is the protective colonization of the oropharynx by probiotic bacteria. Additionally, the stomach, normally inhospitable to bacteria due to its low pH and quick transit time, becomes an overgrown reservoir of potential pathogens in critical illness due to acid blockade and slowed gut motility [14]. This source community to the lung may be similarly displaced by non-pathogenic bacteria administered as probiotics.

#### Immigration of the lower gastrointestinal tract

Though the gut wall normally tightly contains the massive bacterial biomass within its lumen, gut permeability is increased in critically ill patients [15]. Translocation of gut bacteria to the lung has long been a postulated mechanism behind VAP and lung injury, yet culture-dependent studies in the 1990s were unable to confirm the existence or significance of gut translocation [16]. Recent culture-independent studies, both of ICU patients and animal models, have demonstrated that the lungs of critically ill patients are indeed enriched with gut-associated bacteria, undetected by culture and correlated with intensity of lung inflammation [17, 18]. The composition of the gut microbiome itself is profoundly altered in critically ill patients [19], potentially contributing remotely via an altered source community. Thus, another potential mechanism of benefit of enteric probiotics is the colonization of the lower gastrointestinal tract, minimizing the relative microbial burden and pathogenic potential of gut–lung translocation.

#### Elimination and altered growth conditions

Though the growth conditions of lung bacteria are determined by a variety of environmental factors like nutrient supply, oxygen tension, and pH, one key selective pressure in the lung ecosystem is the host’s arsenal of innate and adaptive immune defenses. A large and growing literature supports the hypothesis that probiotics influence systemic immunity and thus may enhance the host’s ability to kill and clear reproducing lung pathogens as they emerge from within the lung community [20]. Thus, by augmenting microbial surveillance and clearance by bolstering systemic immunity, or similarly by preventing the immune derangements provoked by the gut dysbiosis of critical illness, probiotics may favorably rebalance the elimination and growth conditions of the lower respiratory tract.

Finally, probiotics may have potential “off-target” benefits that decrease the incidence or severity of VAP by decreasing the overall severity of the illness and the need for mechanical ventilation. The increasing body of evidence showing the contribution of the microbiome to the pathogenesis of sepsis and multiorgan failure [9], as well as the efficacy of selective gut microbiome *suppression* in improving ICU outcomes, i.e., via selective decontamination of the digestive tract [6, 21], provides a rationale that probiotics may plausibly improve non-VAP outcomes such as shock and respiratory failure, thus shortening the duration of invasive ventilation and decreasing the opportunity for VAP to develop.

Has this strong rationale for the potential benefits of probiotics on VAP incidence and ICU outcomes translated into clinical evidence of its benefits in clinical trials? In a recent landmark trial, the administration of a combined probiotic and prebiotic decreased the incidence of sepsis in infants in rural India [22]. This trial, remarkable both as a positive primary prevention trial for sepsis and as a positive trial for probiotics, has prompted additional interest in the use of probiotics in the prevention and treatment of the diseases of critical illness. We performed a systematic review of all published clinical trials on the efficacy of probiotics to prevent VAP and improve outcome in adult ICU patients.

## Methods

### Search strategy

We conducted an unrestricted search in the databases of MEDLINE via PubMed and Web of Science (the KCI-Korean journal database, the Russian Science Citation Index, and the SciELO Citation Index), using the following keywords: “ventilator–associated pneumonia,” “probiotics,” “therapy,” and “prevention” (see Additional file 1). The reference lists of relevant reviews on this topic were screened for additional potentially relevant articles [23, 24]. The search was finalized in January 2018. Only articles written in English and published after from 2007 till the date of the search were considered for eligibility.

### Inclusion and exclusion criteria

Trials were included if (a) patients were over 18 years of age, (b) patients were admitted to an ICU and receiving invasive ventilation, and (c) when the trial tested a probiotic as a preventive measure. Trials were excluded if they were (a) non-randomized trials; (b) tested another intervention than probiotics, i.e., when probiotics were used in combination with a prebiotic or an antimicrobial agent such as prophylactic antibiotics; and (c) if no data relevant to VAP were reported. There were no restrictions with regard to the definition of VAP, since there is no standard worldwide adopted definition.

### The outcome of interest

The primary outcome was the occurrence of VAP or the incidence of VAP as expressed in the number of VAP episodes per 1000 ventilation days in the intervention and control groups.

### Data extraction

Data were extracted from the eligible articles using a data extraction form developed for this review. Data captured included trial setting and country, trial design, methodological and statistical design, primary and secondary outcome measures, duration of trial, sample size, the tested intervention and the control therapy, and percentage loss to follow-up.

### Risk of bias assessment

The Cochrane Collaboration standardized instrument for assessing the risk of bias was used to assess the methodological quality of the included trials [25].

### Statistical methods

We used Endnote, Microsoft Office Word, and Excel to manage screening and selection of articles. Data were extracted from the included articles using an Excel database. We refrained from doing any meta-analyses because of the heterogeneity in the studied populations, as well as the intervention and the used definitions of VAP.

For each individual study, the relative risk (RR) and its 95% confidence interval (CI) of VAP were calculated using the reported primary outcome data and the formulas below. Formula 1 was used to calculate the relative risk for incidence expressed as a percentage of total patients. Formula 3 was used to calculate the relative risk of VAP for studies where the incidence was expressed as the number of VAP episodes per 1000 ventilator days. In those studies, the 95% confidence interval could not be calculated for the relative risk of this outcome, in the absence of data reporting on the number of non-VAP episodes per 1000 ventilation days.

Formula 1: $\mathrm{Relative}\ \mathrm{risk}=\frac{p_{\mathrm{VAP}\ \mathrm{intervention}\ \mathrm{group}}}{p_{\mathrm{VAP}\ \mathrm{control}\ \mathrm{group}}}$ in which *p* = the incidence of VAP.

Formula 2: $95\%\mathrm{confidence}\ \mathrm{interval}={e}^{\ln \left(\mathrm{RR}\right)\pm 1.96\sqrt{\frac{b}{a\left(a+b\right)}+\frac{d}{c\left(c+d\right)}}}$ in which *a* = number of patients that developed VAP in the intervention group, *b* = number of patients that did not develop VAP in the intervention group, *c* = number of patients that developed VAP in the control group, and *d* = number of patients that did not develop VAP in the control group.

3 $$ \mathrm{Relative}\ \mathrm{risk}=\frac{\mathrm{Number}\ \mathrm{of}\ \mathrm{VAP}\ \mathrm{episodes}/1000\ {\mathrm{ventilator}\ \mathrm{days}}_{\mathrm{intervention}\ \mathrm{group}}}{\mathrm{Number}\ \mathrm{of}\ \mathrm{VAPepisodes}/1000\ {\mathrm{ventilator}\ \mathrm{days}}_{\mathrm{control}\ \mathrm{group}}} $$

## Results

### Search results and trial details

**Fig. 1:**
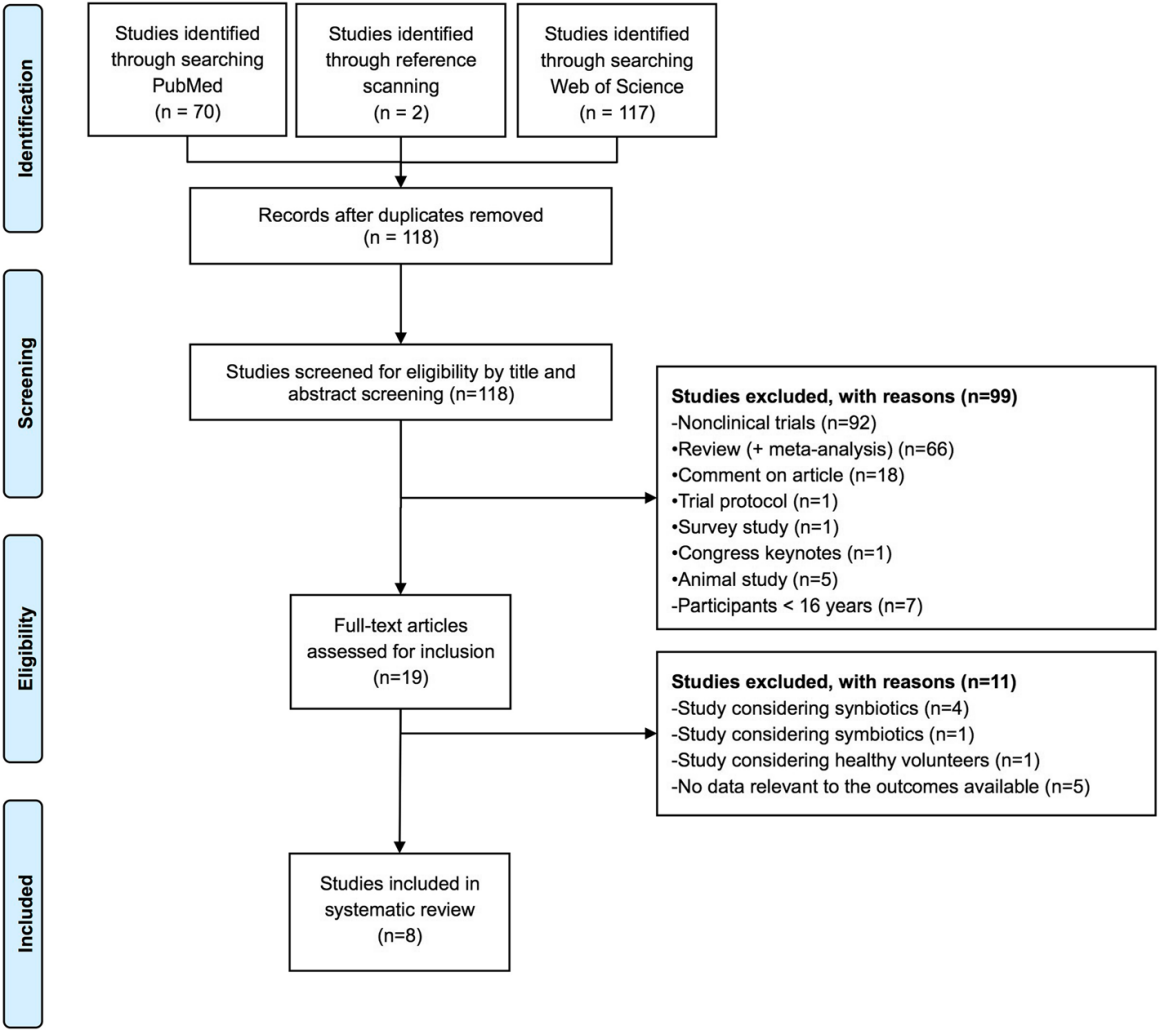
Flowchart outline of the search (only the main reasons of exclusion are displayed)

The search identified a total of 72 articles (Fig. 1). Eight articles enrolling a total of 1229 patients fulfilled the inclusion and exclusion criteria [26–33]. The characteristics of the identified trials are presented in Table 1. Sample sizes ranged from 50 to 300 patients. In four trials, the investigators were blinded for the intervention [26, 27, 29, 33]. Only two trials used an intention-to-treat analysis [26, 29]. Loss to follow-up with regard to the primary endpoint ranged from 0 to 14% in the intervention groups and from 0 to 16% in the control groups. One trial did not specify the definition of VAP [30]. The other trials used predefined diagnostic criteria for VAP, including the presence of infiltrates on a chest radiograph, a change in body temperature (fever or hypothermia), a change in white blood cell counts (leukocytosis or leukopenia), and the presence of purulent sputum or aspirates [26–29, 31–33].

**Table 1:** Overview of the used definitions of VAP

Author, year	Study design	*N* (intervention vs. control)	Analyzed *N* (intervention vs. control)	Details of intervention	Primary outcome
Barraud et al., 2010 [26]	Blinded RCT	87 vs. 80	87 vs. 80	*Bifidobacterium bifidum*, *Lactobacillus acidophilus*, *Lactobacillus casei*, and *Lactobacillus rhamnosus* GG*,* 1/day 2 × 10^10^ CFU in the stomach	28-day mortality
Forestier et al., 2008 [27]	Blinded RCT	118 vs. 118	102 vs. 106	*Lactobacillus casei rhamnosus*, 1/day 1 × 10^9^ CFU in the mouth and stomach	Time of first *Pseudomonas aeruginosa* acquisition
Klarin et al., 2008 [28]	Open-label RCT	25 vs. 25	23 vs. 21	*Lactobacillus plantarum* 299, 2/day 1 × 10^10^ CFU in the mouth	Subsequent samples
Knight et al., 2009 [33]	Blinded RCT	150 vs. 150	130 vs. 129	*Lactobacillus paracasei*, *Lactobacillus plantarum*, *Leuconostoc mesenteroides*, and *Pediococcus pentosaceus*, 2/day 1 × 10^10^ CFU in the stomach	VAP
Morrow et al., 2010 [29]	Blinded RCT	73 vs. 73	68 vs. 70	*Lactobacillus rhamnosus* GG,,2/day 1 × 10^9^ CFU in the oropharynx and stomach	VAP incidence
Rongrungruang et al., 2015 [32]	Open-label RCT	75 vs. 75	75 vs. 75	*Lactobacillus casei* Shirota, 1/day 8 × 10^9^ CFU in the mouth and stomach	VAP
Shinotsuka et al., 2008 [30]	Open-label RCT	16 vs. 12	12 vs. 16	*Lactobacillus johnsonii* La1, 2/day 1 × 10^9^ CFU in the stomach	Colonization of gastrointestinal tract and trachea
Zeng et al., 2016 [31]	Open-label RCT	125 vs. 125	118 vs. 117	*Bacillus subtilis* and *Enterococcus faecalis*, 3/day 9 × 10^9^ in the stomach	VAP

A summary of the risk of bias is detailed in Additional file 1: Figure S1 and S2. The risk of bias was low for two [26, 29], unclear in one [33], but high in the other five trials. Overall, allocation concealment and blinding of participants, personnel, and outcome assessment were at high risk of bias, mostly because investigators were not blind for the intervention in four trials [28, 30–32]. Random sequence generation was adequately described for five trials [26, 27, 29, 30, 33]. Two trials did not report all predefined outcomes [27, 31]. Funding by parties with an interest in an outcome showing a positive trend for the effect of probiotics was declared for three trials [27, 28, 33].

Only three trials reached the predefined sample size [28, 31, 32]. In one trial [26], enrollment of patients was stopped after an unplanned interim analysis was performed because of the distressing results of another trial of probiotics [34], showing that more than 4000 patients per group would be needed to establish a treatment effect. One trial was stopped early because of a low inclusion rate [30]. For the three remaining trials, it remained unclear why the preplanned number of patients was not reached [27, 29, 33].

### Details on the intervention

Seven trials used six different subspecies of *Lactobacillus*: *L. johnsonii* [30], *L. casei* [26, 27, 32], *L. plantarum* [28, 33], *L. rhamnosus* [26, 29], *L. paracasei* [33], and *L. acidophilus* [26] (Table 1), either alone or as a mixture. Other used species were *Pediococcus pentosaceus*, *Leuconostoc mesenteroides*, *Bifidobacterium bifidum*, *Bacillus subtilis*, *and Enterococcus faecalis*. Dosages, route of administration, and dosing schemes varied among the included trials: from 1 × 10^9^ to 2 × 10^10^ CFU per dose; from exclusively through the nasogastric tube to additional application in the mouth or solely in the oropharynx; and one, two, or three times per day (Table 1).

### Incidences of VAP

The incidence of VAP expressed as the percentage of studied patients was reported in seven trials (Table 2). One trial reported 20 episodes in 28 patients but failed to report how many of these episodes involved patients in the probiotics group or in the control group [30]. The incidence of VAP ranged from 4 to 36% in the intervention groups and from 13 to 50% in the control groups. The relative risk for VAP ranged between 0.30 and 1.41 (Table 2). Three trials showed a significant difference in favor of probiotic therapy between the intervention and the control groups [29, 31, 32].

**Table 2:** Incidence of VAP (percentage of total patients and number of VAP episodes/1000 ventilator days)

	Incidence VAP			Incidence VAP		
Author, year	Intervention group (percentage of patients [*n* = patients])	Control group (percentage of patients [*n* = patients])	Relative risk (95% CI)	Intervention group (VAP episodes/1000 ventilator days)	Control group (VAP episodes/1000 ventilator days)	Relative risk
Morrow et al., 2010 [29]	19.1% (*n* = 68)	40.0% (*n* = 70)	0.5 (0.3 to 0.8)			
Rongrungruang et al., 2015 [32]	24.0% (*n* = 75)	29.3% (*n* = 75)	0.8 (0.3 to 0.6)	22.6	30.2	0.8
Klarin et al., 2008 [28]	4.3% (*n* = 23)	14.3% (*n* = 21)	0.3 (0.0 to 2.7)			
Knight et al., 2009 [33]	9.0% (*n* = 130)	13.0% (*n* = 129)	0.7 (0.4 to 1.4)	13.0	14.6	0.9
Forestier et al., 2008 [27]	23.5% (*n* = 102)	22.6% (*n* = 106)	1.0 (0.6 to 1.7)			
Barraud et al., 2010 [26]	26.4% (*n* = 87)	18.7% (*n* = 80)	1.4 (0.8 to 2.5)	23.0	14.6	1.6
Zeng et al., 2016 [31]	36.4% (*n* = 118)	50.4% (*n* = 117)	0.7 (0.5 to 1.0)			

The incidence of VAP expressed as the number of VAP episodes per 1000 ventilation days was reported in three trials (Table 2). The incidence of VAP episodes ranged from 13 to 30 per 1000 ventilation days (13 to 23 VAP episodes per 1000 ventilation days vs. 15 to 30 VAP episodes per 1000 ventilation days in the intervention and the control groups, respectively).

### Side effects

One trial reported diarrhea as a side effect of the intervention [32]. The other six trials did not report on side effects of the intervention.

## Discussion

The results from this systematic review seem to suggest that the administration of probiotics might reduce the incidence of VAP in invasively ventilated ICU patients, but firm conclusions cannot be drawn. There was huge heterogeneity between the trials in terms of the chosen intervention and the definition of VAP. Furthermore, most of the trials were of moderate to poor quality. Based on the available data, probiotics does not seem to be associated with an increase in side effects. The currently available data do not yet support the introduction of probiotics as a preventive measure for VAP, but together with the strong rationale for probiotic based on novel ecological insights, the results justify further evaluation in well-designed randomized clinical trials.

Initial enthusiasm for probiotic therapy in critically ill patients was tempered by the worrisome results from the PROPATRIA trial in patients with acute pancreatitis [34]. In this study, 298 patients with severe acute pancreatitis were randomized between a multispecies probiotic preparation or placebo, applied through a jejunal tube. The trial was stopped early due to a higher mortality in the experimental group. Mortality was explained by bowel ischemia and translocation of gut bacteria to the bloodstream resulting in multiorgan failure. However, the present review does not imply that probiotics are associated with major side effects in a severely ill ICU population without pancreatitis, which is in line with later meta-analysis of trials performed in patients with acute pancreatitis [35]. However, this conclusion on safety should also be read with caution, and safety should be an important endpoint for future studies. The currently available evidence does suggest that the combination of microorganisms and dose of probiotics could be important and that small differences in these factors could result in large differences in effects [35]. In that light, it is quite remarkable that several trials included in this systematic review were positive as the combination of bacterial strains, and the dosage differed considerably between studies. However, it is worth acknowledging that publication bias is common in developing fields and could partly explain this observation. Positive—rather than negative—trials are more likely to get written up, accepted, and published. These all prevented us from estimating an average effect size between the studies and limit strong conclusions on the effectiveness of probiotics in the prevention of VAP.

In addition to heterogeneity, the trials also suffered from poor to moderate methodological quality. There was an especially high probability of selection, performance, and detection bias, which could have turned the results in favor of the intervention. However, higher quality studies did not result in smaller effect sizes, suggesting that bias is not the only explanation for the observed treatment effects. In addition to the above described biases, five out of eight trials did not reach the estimated required sample size. This means that the chance of finding a true clinically significant effect is low. The probability that an observed effect that reached the threshold for statistical significance actually reflects a true effect is lower in low-powered studies, and when they do show positive effects, these tend to be overestimated [36].

So, what should the next trials of probiotics for prevention of VAP look like? Randomized clinical trials of preventive use of probiotics will probably focus on the delivery of a single strain of bacteria in a low dose, based on research in other, more advanced areas [35]. Of note, the trials included in the present systematic review that used a single strain were more likely to report positive results. There are some contexts in which diversity is associated with the disease, e.g., the vaginal microbiome. It remains uncertain whether diversity itself is protective, or just correlates with a healthy or nonpathogenic community composition. It may be that a low-diversity community is fine so long as it is the right taxa. The possibility of adverse effects due to probiotic therapy should also be a focus on new studies. This requires the systematic evaluation of side effects such as culture positivity, also in subgroups that may be more prone to opportunistic infections such as immunocompromized patients. Future trials should also improve other methodological issues. As explained above, reaching the predefined sample size is important to gain a higher confidence in positive results and to limit the possibility of obtaining false-negative results. Another important problem with studies of VAP is the competitive risk of mortality during invasive ventilation. Any analysis on VAP incidence is inherently biased by the fact that an intervention might result in (1) earlier or later mortality or (2) shorter or longer duration of invasive mechanical ventilation. Competitive risk proportional hazard models are the only correct statistical solution for this problem and should be implemented in future investigations [4, 37]. Of note, this is in sharp contrast to any survival analysis, which should focus at the absolute rate of the primary outcome at a certain moment in time, which is exactly the approach the included studies used [38]. Last but not least, it should be acknowledged that the definitions of VAP are not well established and that there is a lot of room per personal interpretation, especially in the evaluation of chest radiography.

In this review, we tried to put a systematic review of probiotic use for the prevention of VAP in an ecological perspective. The major strength of the study is that we used a standardized methodological approach in combination with the newest pathophysiological rationales for the interpretation of the results. Several limitations should also be noted; we could not pool the data from individual studies due to heterogeneity. Strong conclusions could not be reached due to limitations in the methodology of the original studies. Data on ICU length of stay and prevention of antimicrobial resistant pathogen carriage were not systematically assessed in the included studies. Finally, we could not assess the existence of selective reporting.

## Conclusion

The incidence of VAP tended to be lower in patients treated with probiotics in several of the trials identified by the systematic search, but firm conclusion cannot be drawn due to the heterogeneity of the studies and the low quality of evidence. Application of probiotics for the prevention of VAP seems to be safe with few side effects reported in the selected trials, but this also needs to be confirmed. Well-designed and sufficiently powered clinical trials to further evaluate this promising intervention are now warranted.

## Additional file

Additional file 1: A summary of the risk of bias. (DOC 197 kb)

## Abbreviations

RR: Relative risk
SDD: Selective digestive tract decontamination
SOD: Selective oral decontamination
VAP: Ventilator-associated pneumonia
