# Supplementary material for: Manipulation of the microbiome in critical illness—probiotics as a preventive measure against ventilator-associated pneumonia
Source: Intensive Care Med Exp. 2019 Jul 25;7(Suppl 1):37. doi: 10.1186/s40635-019-0238-1 (PMC6658628; doi:10.1186/s40635-019-0238-1)

Supplement with:

**Manipulation of the Microbiome in Critical Illness – Probiotics as a Preventive Measure against Ventilator–associated Pneumonia**

Marel C.E. van Ruissen^1^, Lieuwe D. Bos^2,3^, Robert P. Dickson^4^, Arjen M. Dondorp^3,5^, Constance Schultsz^1^, and Marcus J. Schultz^3,5,6^

^1^Amsterdam Institute for Global Health and Development (AIGHD), Academic Medical Center, Amsterdam, the Netherlands

^2^Department of Pulmonology, Academic Medical Center, Amsterdam, the Netherlands

^3^Department of Intensive Care, Academic Medical Center, Amsterdam, the Netherlands

^6^Laboratory of Experimental Intensive Care and Anesthesiology (L·E·I·C·A) , Academic Medical Center, Amsterdam, the Netherlands

^4^Department of Internal Medicine, Division of Pulmonary and Critical Care Medicine, University of Michigan Medical School, Ann Arbor, Michigan

^5^Mahidol–Oxford Tropical Medicine Research Unit (MORU), Mahidol University, Bangkok, Thailand

**Correspondence:**

Prof. Marcus J. Schultz, MD PhD

Academic Medical Center

C3-425

Department of Intensive Care

Meibergdreef 9

1105 AZ Amsterdam

The Netherlands

Email: [marcus.j.schultz@gmail.com](mailto:marcus.j.schultz@gmail.com)

**Supplement** **Methods**

*Search strategy*

(((((((((((("Pneumonia, Ventilator-Associated"[Mesh]) OR "ventilator associated pneumonia"[All Fields]) OR "pneumonia, ventilator associated"[All Fields]) OR "ventilator-associated pneumonia"[All Fields]) OR "ventilator induced lung injury"[All Fields]) OR "VAP"[All Fields])) OR (("pneumonia"[All Fields]) AND "ventilator-associated"[All Fields])) OR (("pneumonia"[All Fields]) AND "ventilator associated"[All Fields])) OR ((("ventilator"[All Fields]) AND "associated"[All Fields]) AND "pneumonia"[All Fields])))

**AND**

((((((((((("Probiotics"[Mesh]) OR "probiotics"[All Fields]) OR "probiotic bacterium"[All Fields])) OR (("probiotic"[All Fields]) AND "bacterium"[All Fields])) OR "probiotic bacteria"[All Fields]) OR (("probiotic"[All Fields]) AND "bacteria"[All Fields])) OR "probiotic flora"[All Fields]) OR (("probiotic"[All Fields]) AND "flora"[All Fields])) OR "probiotic microflora"[All Fields]) OR (("probiotic"[All Fields]) AND "microflora"[All Fields])))

**AND**

(((((((((((("prevention and control"[Subheading]))) OR (("prevention"[All Fields]) AND "control"[All Fields])) OR (((("prevention and control"[All Fields])) OR "prevention"[All Fields]) OR "preventive therapy"[All Fields])) OR (("preventive"[All Fields]) AND "therapy"[All Fields])) OR ((("prophylaxis"[All Fields]) OR "preventive measures"[All Fields]) OR "preventive measure"[All Fields])) OR (("preventive"[All Fields]) AND "measures"[All Fields])) OR (("preventive"[All Fields]) AND "measure"[All Fields])) OR "control"[All Fields]))

OR ((((((((((("therapy"[Subheading]) OR "therapy"[All Fields]) OR "treatment"[All Fields]) OR "treatments"[All Fields]) OR "therapeutics"[MeSH Terms]) OR "therapeutics"[All Fields]) OR "therapeutic"[All Fields]) OR "therapies"[All Fields]) OR "therapeutic use"[All Fields])) OR (("therapeutic"[All Fields]) AND "use"[All Fields])))

**Supplement Results**

Additional file 1, Figure S1 Risk of bias per domain for all studies (
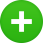
 = low risk,
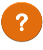
 = unclear risk,
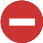
 = high risk)

|  | Random sequence generation (selection bias) | Allocation concealment (selection bias) | Blinding of participants and personnel (performance bias) | Blinding of outcome assessment (detection bias) | Incomplete outcome data addressed (attrition bias) | Selective reporting (reporting bias) | Other sources of bias |
| --- | --- | --- | --- | --- | --- | --- | --- |
| Morrow et al., 2010 | 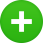 | 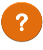 | 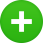 | 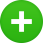 | 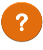 | 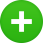 | 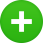 |
| Rongrungruang et al., 2015 | 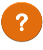 | 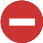 | 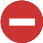 | 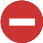 | 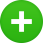 | 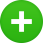 | 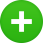 |
| Klarin et al., 2008 | 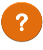 | 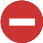 | 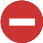 | 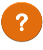 | 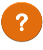 | 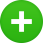 | 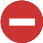 |
| Knight et al., 2008 | 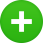 | 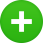 | 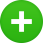 | 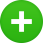 | 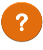 | 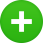 | 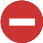 |
| Forestier et al., 2008 | 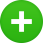 | 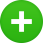 | 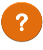 | 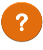 | 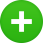 | 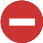 | 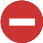 |
| Barraud et al., 2010 | 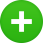 | 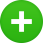 | 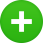 | 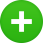 | 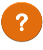 | 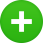 | 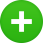 |
| Zeng et al., 2016 | 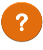 | 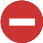 | 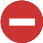 | 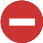 | 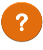 | 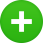 | 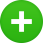 |
| Shinotsuka et al., 2008 | 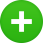 | 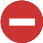 | 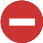 | 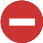 | 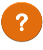 | 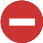 | 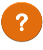 |

Additional file 1, Figure S2. Risk of bias per domain for the individual studies (
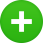
 = low risk,
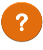
 = unclear risk,
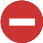
 = high risk)


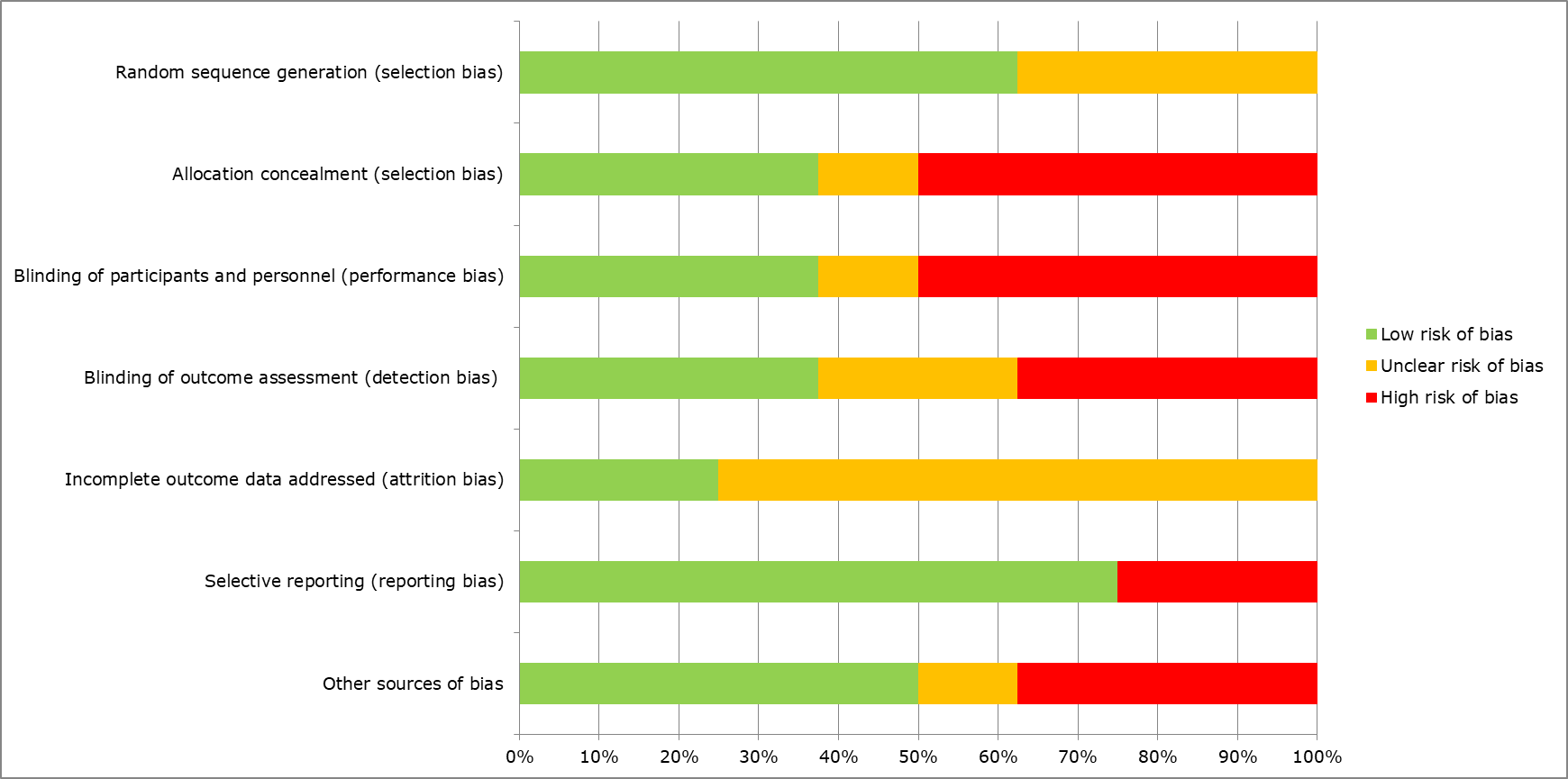

Supplement: Supplementary file 1 — A summary of the risk of bias. (DOC 197 kb) [file 40635_2019_238_MOESM1_ESM.doc]
